# Neutrophils’ Contribution to Periodontitis and Periodontitis-Associated Cardiovascular Diseases

**DOI:** 10.3390/ijms242015370

**Published:** 2023-10-19

**Authors:** Barbara Bassani, Martina Cucchiara, Andrea Butera, Omar Kayali, Alessandro Chiesa, Maria Teresa Palano, Francesca Olmeo, Matteo Gallazzi, Claudia Paola Bruna Dellavia, Lorenzo Mortara, Luca Parisi, Antonino Bruno

**Affiliations:** 1 Laboratory of Innate Immunity, Unit of Molecular Pathology, Biochemistry and Immunology, Istituto di Ricovero e Cura a Carattere Scientifico (IRCCS) MultiMedica, 20138 Milan, Italy; barbara.bassani@multimedica.it (B.B.); martina.cucchiara@multimedica.it (M.C.); omar.kayali@multimedica.it (O.K.); mariateresa.palano@multimedica.it (M.T.P.); francesca.olmeo@multimedica.it (F.O.); matteo.gallazzi@multimedica.it (M.G.); 2 Immunology and General Pathology Laboratory, Department of Biotechnology and Life Sciences, University of Insubria, 21100 Varese, Italy; lorenzo.mortara@uninsubria.it; 3 Unit of Dental Hygiene, Section of Dentistry, Department of Clinical, Surgical, Diagnostic and Pediatric Sciences, University of Pavia, 27100 Pavia, Italy; andrea.butera@unipv.it (A.B.); alessandro.chiesa@unipv.it (A.C.); 4 Department of Biomedical, Surgical and Dental Sciences, University of Milan, 20122 Milan, Italy; claudia.dellavia@unimi.it

**Keywords:** periodontitis, neutrophils, dental disorders, diabetes, atherosclerosis

## Abstract

Neutrophils represent the primary defense against microbial threats playing a pivotal role in maintaining tissue homeostasis. This review examines the multifaceted involvement of neutrophils in periodontitis, a chronic inflammatory condition affecting the supporting structures of teeth summarizing the contribution of neutrophil dysfunction in periodontitis and periodontal-related comorbidities. Periodontitis, a pathological condition promoted by dysbiosis of the oral microbiota, is characterized by the chronic inflammation of the gingiva and subsequent tissue destruction. Neutrophils are among the first immune cells recruited to the site of infection, releasing antimicrobial peptides, enzymes, and reactive oxygen species to eliminate pathogens. The persistent inflammatory state in periodontitis can lead to aberrant neutrophil activation and a sustained release of proinflammatory mediators, finally resulting in tissue damage, bone resorption, and disease progression. Growing evidence now points to the correlation between periodontitis and systemic comorbidities. Indeed, the release of inflammatory mediators, immune complexes, and oxidative stress by neutrophils, bridge the gap between local and systemic immunity, thus highlighting neutrophils as key players in linking periodontal inflammation to chronic conditions, including cardiovascular diseases, diabetes mellitus, and rheumatoid arthritis. This review underscores the crucial role of neutrophils in the pathogenesis of periodontitis and the complex link between neutrophil dysfunction, local inflammation, and systemic comorbidities. A comprehensive understanding of neutrophil contribution to periodontitis development and their impact on periodontal comorbidities holds significant implications for the management of oral health. Furthermore, it highlights the need for the development of novel approaches aimed at limiting the persistent recruitment and activation of neutrophils, also reducing the impact of periodontal inflammation on broader health contexts, offering promising avenues for improved disease management and patient care.

## 1. Introduction

### 1.1. Introduction to the Biology of Neutrophils

Neutrophils are polymorphonuclear leukocytes and professional phagocytes of the innate immune system, playing a crucial role as the first line of defense in response to invading pathogens. As the most abundant type of white blood cells, neutrophils constitute approximately 50–70% of the overall circulating leukocytes, with a life span demonstrated to be up to 5 days [1,2,3].

Besides acute inflammation, neutrophils emerge as crucial players during chronic inflammation, autoimmunity, and tumors also due to their direct (cell–cell contact) or indirect (via soluble factors) crosstalk with other cells of both innate and adaptive immunity, including macrophages, dendritic cells, and T-cells [4].

The development of neutrophils, also known as granulopoiesis, occurs within the bone marrow (BM), through a fine-tuned multistep developmental process which starts with a common multipotent hematopoietic stem cell (HSC) [5]. During hematopoiesis, HSCs lose their self-renewal potential and produce multipotent precursors (MPPs) which are then committed toward the common lymphoid and myeloid precursors (LMPs) upon a high transcriptional level of PU.1. Subsequently, LMPs differentiate into granulocyte-monocyte precursors (GMPs) upon granulocyte colony-stimulating factor (G-CSF) stimulation [6]. The activation of the transcription factor family of CCAAT enhancer-binding protein alpha (C/EBPα), among other key molecular components, plays a crucial role in both initiating neutrophil development from GMPs and promoting their terminal differentiation [7]. Some of the neutrophil precursors give rise to a promyelocyte pool. The earliest unipotent neutrophil progenitors (eNePs) differentiate in the bone marrow to generate immature (banded) neutrophils and mature neutrophils. In physiological conditions, only mature neutrophils enter the circulation, while in chronic diseases, including cancer, immature neutrophils, as well as neutrophil progenitors, expand in the bone marrow and are released into the circulation [8].

During inflammation or infection, specific neutrophil chemo-attractants (e.g., tumor necrosis factor-alpha (TNF-α), interleukin-1 beta (IL-1β), IL-17, leukotrienes, prostaglandins, complement component C5a [7], and bacteria products such as peptidoglycan and phenol-soluble modulins) are released from the inflamed tissue [9], driving mature neutrophil homing via postcapillary venules, in a fashioned process known as neutrophil recruitment [10].

### 1.2. Neutrophil Homeostasis and Activation

Neutrophils are recruited from the blood to the site of infection through a multistep cascade that is termed “extravasation”. Resident damaged cells can release several factors, including cytokines and chemokines, that induce the expression of E-selectins on the surface of endothelial cells interacting with sialyl-Lewis^X^ present on neutrophils. The low-affinity interaction between selectins and sialyl Lewis^X^ mediates neutrophils rolling onto the endothelium [11]. After chemotactic stimuli, a stronger bind mediated by leukocyte integrins, and their ligands expressed by endothelial cells determines the firm adhesion of neutrophils. Thereafter, neutrophils adopt a polarized shape and crawl onto the endothelial apical surface to extravasate and migrate in the site of infection [11]. The locomotion of leukocytes is strictly dependent on β2 integrins. Following the resolution of infection, in homeostatic conditions, neutrophils leave the site of inflammation, generating the so-termed “reverse migration”. This phenomenon, first described by Mathias et al. in 2006 [12], refers to the ability of neutrophils to move away from the site of inflammation back into the vascular system, allowing for the resolution of infection and preventing damages to surrounding tissues due to persistent neutrophil activation [13].

The primary function of neutrophils lies in their ability to efficiently recognize, engulf, and eliminate microorganisms through phagocytosis. Neutrophils use a wide range of pattern recognition receptors (PRRs), such as toll-like receptors (TLRs), or the deposition of opsonins, such as complement and antibodies on the pathogen’s surface, that enable them to detect and bind to microbial components, initiating the phagocytic process [1,2,3].

Phagocytosis requires the formation of a defined vacuole known as the phagosome that is sustained by the actin–myosin contractile system. The phagosome subsequently fuses with a lysosome to form the phagolysosome, a structure containing proteolytic enzymes, antimicrobials, such as defensins, and lactoferrin, and characterized by the production of reactive oxygen species (ROS), including superoxide anion, hydrogen peroxide, and hydroxyl radicals by NADPH oxidase, in a process termed *oxidative.* (*respiratory*) *burst*, which allows for the elimination of internalized pathogens [9]. Alternatively, neutrophils can be activated in response to various signals, including pathogen-associated molecular patterns (PAMPs) or damage-associated molecular patterns (DAMPs) that bind cell surface receptors. Their activation leads to the release of different enzymes (such as myeloperoxidase (MPO), elastase (NE), cathepsin G) contained in specialized granules in the extracellular space, ultimately resulting in the elimination of the pathogens. This process is known as neutrophil degranulation [2,9,14,15].

In addition to their direct antimicrobial functions, neutrophils can release proinflammatory cytokines, including TNF-α and IL-1β, which promote local inflammation and recruit other immune cells to the site of infection [2,9,14,15].

To eliminate microorganisms, neutrophils can also generate peculiar structures, namely neutrophil extracellular traps (NETs), in a process termed NETosis [16].

### 1.3. Neutrophil Extracellular Traps (NETs)

NETs were discovered relatively recently in the field of immunology when Brinkmann and Zychlinsky, in 2004, demonstrated that neutrophils, stimulated with phorbol myristate acetate (PMA), were able to release web-like structures composed of cytosolic and granule proteins, such NE, MPO, and cathepsin G, assembled on a scaffold of DNA. These structures were first identified as a mechanism through which neutrophils can exert their antimicrobial effects, immobilizing and killing bacteria extracellularly [16]. The release of NET was originally associated with neutrophil death. Indeed, Fuchs and colleagues, describing the process of NETosis, showed that it relied on the lysis of the nuclear envelope, which occurred upon chromatin decondensation and the mingling of chromatin with granule proteins within the cytoplasm. This results in cytoplasmic membrane lysis and NET release in the extracellular space [17]. As such, NETosis was initially considered as a cell death program, distinct from apoptosis or necrosis. This process is finely tuned by several proteins, and it is strictly dependent on the generation of ROS. It has been shown that PMA stimulation results in the activation of nicotinamide adenine dinucleotide phosphate (NADPH) oxidase, via the PKC and Raf-MEK-ERK signaling pathway, which contributes to ROS generation [18]. The production of ROS, on one hand, activates protein arginine deiminase 4 (PAD4), which catalyzes the citrullination/deamination of arginine residues of histones, thereby promoting chromatin decondensation [19,20] and, on the other hand, induces the release of MPO and NE from cytoplasmic azurophilic granules. NE activates gasdermin D [21] which supports the formation of pores in the plasma membrane and granule membranes, enhancing the release of NE and other granule content. Therefore, the nuclear envelope breaks down via cell-cycle proteins [22] releasing chromatin in the cytosol, which mixes with cytosolic and antimicrobial proteins.

Besides this process, which has been termed “lytic NETosis” or “suicidal NETosis” [23], the concept of “vital NETosis”, a NADPH oxidase-independent process in which the extrusion of NET does not necessarily result in cell death, has recently been introduced [24]. Mechanistically, it has been found that nonlytic NET release occurs via toll-like receptor (TLRs) signaling [24,25], which leads to PAD4 [26] activation and chromatin decondensation. However, instead of plasma membrane disruption, chromatin is secreted using vesicles, hence preserving neutrophil integrity.

In addition, human neutrophils primed with granulocyte–macrophage colony-stimulating factor and subsequently stimulated with LPS or complement factor 5a have been observed to release NETs of mitochondrial origin (mtDNA), which are composed of mitochondrial, instead of nuclear, DNA [27]. Since the molecular mechanisms that underly mtDNA NET formation have not been fully elucidated, recently, Amini and colleagues demonstrated the crucial role of optic atrophy 1 (OPA1) in this process, showing that the genetic deletion of OPA1 in neutrophils limits defects in antibacterial activity due to their inability to form NETs [28].

### 1.4. NETs in Diseases

Even if neutrophils are crucial for host defense, persistent neutrophil activation leads to excessive inflammation, in turn resulting in surrounding tissue damage that contributes to the pathogenesis of inflammatory diseases.

The exacerbated activation of neutrophils has been observed in diverse conditions spanning from autoimmune diseases to chronic obstructive pulmonary disease (COPD), sepsis, as well as periodontitis [24]. The shared features of neutrophil hyperactivity point to the relevance of these cells in promoting inflammation-related diseases. So far, NETs have been identified in the lungs in patients with cystic fibrosis (CF), acute lung injury (ALI), and allergic asthma [29]. Moreover, an increase in circulating cell-free DNA (cfDNA) characterizes autoimmune diseases, including vasculitis, systemic lupus erythematosus (SLE), and rheumatoid arthritis (RA).

NETs have been associated with the pathogenesis of SLE due to their potential role in exposing self-antigens, thus triggering the formation of autoantibodies [30]. Moreover, NETs can activate plasmacytoid dendritic cells to produce high levels of IFN-α, resulting in capillary and renal cell necrosis and podocyte loss [30]. In the context of RA, persistent NET formation leads to the production of deaminated antigens (i.e., citH2A, citH2B, and citH4), which leads to the generation of autoantibodies and, in turn, results in the inflammation of synovial membranes [31]. NETs can also promote the formation of blood clots, providing the scaffold for platelet adhesion and activation and causing thrombosis and strokes. In line with this, the detection of high plasma NET-related biomarkers correlates with worse stroke outcomes [32].

Conflicting results have been obtained in cancer since NETs have been associated with both the promotion and inhibition of cancer. On the one hand, they can trap and kill cancer cells, limiting tumor growth and dissemination [33,34]. On the other hand, they may exert protumor functions, creating a proinflammatory environment and promoting angiogenesis and metastasis [14,35,36].

In the context of periodontitis, NETs and their associated proteins have been observed either in tissue lesions or in the blood of patients with common/chronic forms of periodontal disease [37,38].

In 2021, Silva and colleagues demonstrated that fibrin, through the α_M_β_2_ integrin receptor, promotes the activation of neutrophils and the release of NETs, significantly contributing to bone loss around the teeth [39].

More recently, the same group better clarified the cellular mechanisms involved in NET-related periodontitis, showing that neutrophils are among the first immune cells to infiltrate the gingival and oral mucosa after disease induction and are crucial in enhancing inflammatory-associated conditions. Indeed, neutrophil depletion using neutralizing antibodies significantly reduced inflammatory bone destruction. The authors also showed a marked production of NETs in periodontal lesions, demonstrating that extracellular histones can promote T-cell differentiation toward Th17, resulting in bone destruction [40,41].

### 1.5. Neutrophils and Trained Immunity

The identification of different PRRs, such as TLRs, NOD-like receptors, RigI helicases, and C-type lectin receptors that trigger the activation of innate immune cells in response to PAMPs and DAMPs paved the way for the introduction of trained immunity [42]. This concept was first proposed by Netea in 2011 and describes the immunological memory response that characterizes innate immune cells in response to past insults [43]. Indeed, the assumption that innate immune cells can exhibit a memory is widely accepted in plant immunology and is strongly suggested in invertebrates, as well as showing a potential application also in vertebrate immunology [43]. Following their exposure to specific stimuli, innate immune cells can mold an enhanced response to previously encountered infectious agents. This challenging concept was empirically confirmed in 2012 by Kleinnijenhuis et al., that demonstrated that bacillus Calmette-Guérin (BCG) vaccination in healthy subjects resulted not only in an increased production of IFN-γ, but also in an enhanced TNFα and IL-1β release by monocytes in response to unrelated bacterial and fungal pathogens, persisting also 3 months after vaccination [44]. In immunodeficient mice lacking T and B lymphocytes, BCG vaccination against Mycobacterium tuberculosis conferred crossprotection and reduced mortality caused by a lethal Candida albicans infection, thus suggesting the induction of a nonspecific protection from infections due to a reprogramming of innate immune cells [44]. Unlike the classical immunological memory of adaptive immunity involving gene rearrangement and the clonal expansion of antigen-specific lymphocytes, trained immunity is mainly due to epigenetic modulation and metabolic reprogramming that modify transcriptional programs and functions of innate immune cells and increase their response to secondary challenges. Altered chromatin accessibility in trained immune cells or their precursors has been observed by transposase-accessible chromatin with high-throughput sequencing (ATAC-seq) [45], suggesting that trained immunity can represent an altered functional state of the stimulated cell that persists for weeks to months.

Given these data, several studies focused their attention on trained immunity and the molecular mechanisms that govern the acquisition of memory in different innate immune cells, including monocytes, natural killer cells, and, more recently, neutrophils. Indeed, while trained immunity is a concept that has been more extensively studied in other innate immune cells, there is evidence pointing out that some aspects of trained immunity can be also acquired by neutrophils.

This was clearly demonstrated by Kalafati and coworkers, who showed that the pretreatment of mice with a trained immunity agonist, namely β-glucan, limited tumor growth [45]. The antitumor effect of β-glucan-induced trained immunity was associated with transcriptomic changes in granulocytes, which are skewed toward an antitumor phenotype, characterized by an upregulation of genes involved in phagocytosis, antigen presentation, migration, and ROS production. The authors, using single-cell ATACseq on splenic neutrophils and bone marrow GMPs from mice treated with β-glucan, also observed epigenetic modifications in regions involved in the regulation of myeloid cell differentiation and cell metabolism [45].

Moreover, it has been shown that BCG vaccination of healthy humans induces long-lasting changes in neutrophil phenotype, characterized by the upregulation of activation markers, such CD11b and CD66b, and decreased expression of CD62L and PD-L1 [46]. BCG vaccination significantly increased the production of IL-8 and NE upon ex vivo exposure to secondary pathogenic stimuli. Also, increased ROS production, phagocytosis, and enhanced expression of degranulation markers were observed in neutrophils exposed to unrelated pathogens/stimuli, highlighting the promotion of antimicrobial function [46]. The enhanced killing abilities of human neutrophils persisted for 90 days after vaccination and were associated with the activation of the MAPK pathway, with an increased phosphorylation of MAPK-activated protein kinase 2 (pMAPKAPK2), cAMP-response element binding protein (CREB), and chromatin remodeling. BCG vaccination induced epigenetic modifications, increasing H3K4me3 at the promoter sites of genes involved in antimicrobial function, including signal transducer and activator of transcription 4 (STAT4) [46].

Even if there are no empirical data demonstrating a link between periodontitis and HSPC training, an interesting association has recently been pointed out by Hajishengallis and colleagues [47]. Indeed, the authors speculated that the systemic inflammation promoted by periodontitis may contribute to shaping an inflammatory bone marrow microenvironment, resulting in the modulation of hematopoietic stem and progenitor cells promoting increased myelopoiesis. This might lead to the generation of hyper-responsive or hyperinflammatory myeloid cells that can be recruited to the sites of infection, thus exacerbating periodontitis and supporting a self-sustained feed-forward loop that could contribute to the chronicity of the oral condition [47]. This assumption is sustained by a recent study which showed that periodontal-associated inflammation positively correlated with both bone marrow activity and arterial inflammation. A significant association was observed between hematopoietic activity and inflammatory biomarkers, such as C-reactive protein levels, white blood cell count, and monocyte count [48].

In mouse models, it has been shown that the ligature-induced periodontitis procedure is associated with an increased frequency of bone marrow neutrophils and with an enhanced neutrophil response to subsequent acute infectious peritonitis, suggesting that periodontitis significantly impacts the severity of other inflammatory conditions [49].

## 2. Periodontitis

Periodontitis is a common and potentially severe oral health condition characterized by the inflammation and infection of tissues surrounding the teeth [50]. Periodontitis is classified as a progressive disease that, if left untreated, can lead to tooth loss, and have adverse effects on overall health [50,51]. A major etiological agent in periodontitis is the accumulation of dental plaque, a sticky film of bacteria, food particles, and saliva, on the teeth and gums [50,51,52]. Other relevant contributing factors to periodontitis include poor oral hygiene, smoking habits, presence of comorbidities, and inadequate medications that support bacterial growth and increase the risk of periodontal disease [50,51,52].

Diverse therapeutic options are employed in the management of periodontitis and include scaling and root planning, use of antibiotics to limit local microbial infection and possible bacterial spread, and, in advanced cases, surgical procedures or bone grafting necessary to repair damage and promote tissue regeneration.

Concerning the management of periodontitis with conventional antibiotics, it has been demonstrated that this option does not appear to be effective in the absence of mechanical debridement. In this context, the use of antimicrobial peptides and proteins has emerged as next-generation approach to the management of periodontitis, as exhaustively discussed in [53].

As an inflammatory disease of the oral mucosa, periodontitis is epidemiologically associated with other chronic inflammation-driven disorders, including cardio-metabolic, neurodegenerative, and autoimmune diseases and cancer [48,49,54,55,56,57,58,59,60].

Inflammation has been characterized as a major host-dependent hallmark of chronic-degenerative diseases, such as pathologies affecting the cardiovascular system [57,61,62,63,64,65]. In this scenario, chronic inflammation of the periodontium results in the continuous and uncontrolled release of cytokines and prostaglandins, bacterial toxins that cooperate in the generation of an altered and over-immune-stimulated micro- (tissue-local) and macro- (circulation) environment [66,67]. This overstimulation contributes to the generation of relevant damage to the cardiovascular system, which is constantly exposed to cytokines and ROS that further contribute to the exacerbation of the chronic inflammatory state [68,69]. Also, nonsevere periodontitis is associated with low-grade inflammation, which is a relevant etiological agent for cardiovascular diseases (CVD) [67].

## 3. Neutrophils, Oral Cavity, and Periodontitis

The oral cavity represents a unique environment characterized by dynamic and ever-changing microbiota. Despite this dynamic nature, homeostatic mechanisms exist to maintain a balanced interaction between the oral microbiota and the immune system. Notably, neutrophils play a pivotal role in this equilibrium by being actively recruited into the gingival sulcus through the continuous secretion IL-8/CXCL8 gradient by the junctional epithelium. This recruitment is crucial as the oral biofilm, which comprises a diverse bacterial community, intimately interacts with the junctional epithelium. In this context, neutrophils’ primary functions are to regulate the biofilm and ensure periodontal health [70].

**Figure 1:**
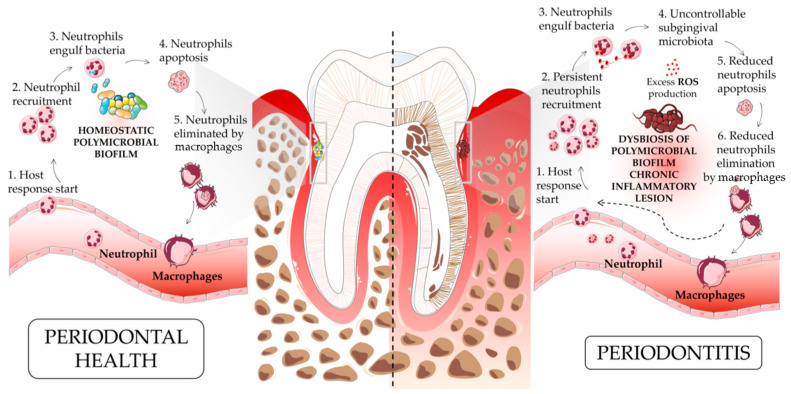
**An overview of the pathogenesis of periodontitis in a neutrophil-driven fashion.** The acute inflammatory process (angiopholgosis) is initiated by the infiltration of leukocytes, recruited to limit bacterial invasion. Proresolution mediators, induced as a mechanism of host defense, downregulate the recruitment of immune cells and the uptake of apoptotic neutrophils by macrophages to facilitate the clearance of the inflammatory lesion (adequate balance host defense). Periodontitis is characterized by both the dysbiosis of oral microbiota and a persistent proinflammatory state due to the reduced ability to eliminate pathogens, followed by an unresolved lesion state that leads to the immune-cell-mediated self-destruction of periodontal tissues, which results in the chronicization of periodontitis (inadequate host defense balance).

However, during gingivitis, a state of moderate inflammation occurs. If this inflammatory response remains uncontrolled, such as in cases of poor oral hygiene, gingivitis can progress to periodontitis. In periodontitis, neutrophils fail to effectively eliminate or control microbial pathogens. As a result, there is an increased recruitment of neutrophils into periodontal tissue (Figure 1). Paradoxically, the accumulation of neutrophils, instead of protecting periodontal tissues, contributes to tissue damage and potential bone loss. Thus, maintaining a delicate balance between neutrophil function and microbial challenge is crucial for ensuring periodontal health (Figure 1).

Neutrophils have been found as abundant innate immune cells present in the periodontal tissue [71], gingival crevicular fluid, and oral cavity [72]. Neutrophil infiltration has also been observed in edentulous patients, supporting their capability to transmigrate the oral mucosa directly [72]. CD177^+^ neutrophils have been found within supragingival dental biofilms, playing different roles in periodontitis.

During their migration process, neutrophils leave the capillary network of the underlying connective tissue and pass through the junctional epithelium into the crevicular space, where they accumulate at the level of the subgingival plaque and the gingival epithelium [73]. Their migration from local capillaries is driven by an IL-8/CXCL8 chemotactic gradient [73]. There is still a matter of debate on whether neutrophil recruitment into the crevice accounts for the healthy maintenance of periodontal tissue as a physiological process or as a basic inflammatory process.

### 3.1. Neutrophils and Periodontitis

Periodontitis is a chronic inflammatory disease initiated by the dysbiosis of microbial communities in dental plaque and exacerbated by an enhanced host immune response. The infiltration of neutrophils into periodontal tissues represents one of the hallmarks of periodontitis, indicating an active immune response against periodontal pathogens. However, the prolonged presence and excessive activation of neutrophils can contribute to tissue damage and destruction in the periodontium. The recruitment of neutrophils to periodontal tissues is orchestrated by a complex interplay of chemokines, cytokines, and adhesion molecules. These molecules are upregulated in response to microbial challenges, leading to the migration of neutrophils from the bloodstream into periodontal tissues. Factors such as *Porphyromonas gingivalis*, *Aggregatibacter actinomycetemcomitans*, and lipopolysaccharides (LPS) derived from periodontal pathogens contribute to the enhanced recruitment and activation of neutrophils in the periodontium [73].

Patients diagnosed with periodontitis display increased neutrophil counts and phenotypes characterized by hyper-reactive cellular functions [74,75]. These alterations encompass the heightened production of ROS, in response to fMLP, Phorbol 12-Myristate 13-Acetate PMA, or periodontal pathogens [21,22,23,76,77,78,79,80]; increased TNF-α production upon stimulation with the periodontal pathogen *Fusobacterium nucleatum* in vitro and elevated levels of neutrophil elastase, associated with periodontal tissue damage. The systemic impact of these changes was recently corroborated by an experimental study employing mice with experimental periodontitis, which exhibited an exaggerated inflammatory neutrophil response when exposed to secondary peritonitis compared to periodontitis-free mice [81]. Activated neutrophils release a variety of antimicrobial substances, including reactive oxygen species (ROS), proteases, and antimicrobial peptides. ROS production by neutrophils plays a dual role in periodontitis, as it can efficiently kill bacteria but also contributes to tissue damage through oxidative stress. Neutrophil-derived proteases, such as matrix metalloproteinases (MMPs), elastase, and cathepsins, degrade extracellular matrix components and contribute to the destruction of periodontal tissues. Moreover, neutrophils release proinflammatory cytokines, further amplifying the local immune response and exacerbating tissue damage [82].

Neutrophils engage in intricate interactions with periodontal pathogens. While neutrophils initially aim to eliminate pathogens, certain periodontal bacteria can evade neutrophil-killing mechanisms and even manipulate neutrophil functions to their advantage. For instance, *Porphyromonas gingivalis* can limit neutrophil chemotaxis and phagocytosis, leading to impaired bacterial clearance and prolonged inflammation. Moreover, the formation of NETs may contribute to periodontal tissue destruction, as NETs can promote inflammatory responses and contribute to the release of proinflammatory mediators.

### 3.2. Neutrophil Extracellular Traps and Periodontitis

NETs were firstly described by Brinkmann et al. as bactericidal structures that play a crucial role in disarming and eliminating extracellular bacteria [16]. NET formation involves the extrusion of nuclear chromatin into the extracellular milieu by rupturing the nuclear and plasma membranes. The extruded chromatin is intricately associated with proteins derived from cytoplasmic neutrophilic granules, thereby establishing a network capable of capturing and neutralizing pathogens [83]. The extrusion of NETs from dying neutrophils has been implicated in the pathogenic tissue damage observed in periodontal tissues, potentially mediated through an autoimmune response [84]. However, in 2012, Pilsczek et al. introduced an alternative perspective, proposing that neutrophils can generate NETs even in the presence of advanced Staphylococcus aureus infections while retaining their viability and functional capabilities, including intact phagocytic activity and other essential neutrophil functions [85]. NET production and/or effectiveness in periodontitis may be limited, thus allowing easier bacterial infiltration of periodontal tissues, leading to a more inflammatory response in the infected area and resulting in tissue destruction.

Several microorganisms have developed different mechanisms to escape NETs [86]. This includes the production of DNases and other extracellular nucleases, to degrade the DNA backbone of NETs, thus evading this host defense mechanism. Staphylococcus aureus and Streptococcus pneumoniae, bacterial species largely associated with periodontal diseases, have been found to produce DNase as a mechanism to escape NETs [9,87,88]. DNase production has been documented among a wide range of bacterial species associated with periodontal disease, particularly in those traditionally recognized as periodontal pathogens belonging to the red (*Porphyromonas gingivalis* and *Tannerella forsythia*), orange (*Fusobacterium nucleatum*, *Prevotella intermedia*, and *Prevotella nigrescens*), and yellow (*Streptococcus gordonii*) microbial complexes. DNase expression analysis performed on six different strains of *Porphyromonas gingivalis*, a key periodontal pathogen, revealed varying degrees of DNase activity [89]. *Porphyromonas gingivalis* possesses the ability to induce NET formation through the action of gingipains; however, its proteolytic activity has been shown to deactivate the bactericidal components of NETs via the activation of protease-activated receptor-2 [90]. Investigation of mutant and wild-type strains of *Porphyromonas gingivalis* has confirmed the peculiar induction of NET formation by the mutant strains [91]. *Prevotella intermedia* has also demonstrated substantial nuclease activity, compared to other periodontal bacterial species, suggesting its potential role in evading the antimicrobial action of NETs within the biofilm environment. Conversely, *Aggregatibacter actinomycetemcomitans*, another prominent periodontal pathogen, displayed no detectable DNase activity in the same study [92].

## 4. Neutrophils, Periodontitis, and Other Comorbidities

The relationship between periodontitis and cardiovascular disorders has already been highlighted by different studies [93] and is of considerable interest due to its bidirectional nature and the potential interplay and mutual impact on public health of the two conditions.

A recent meta-analysis including twenty-six studies reported that in patients with periodontal disease, the prevalence of cardiovascular disorders was 7.2%, pointing to a significant association between periodontitis and cardiovascular diseases, with no significant differences between men and women [93].

**Figure 2:**
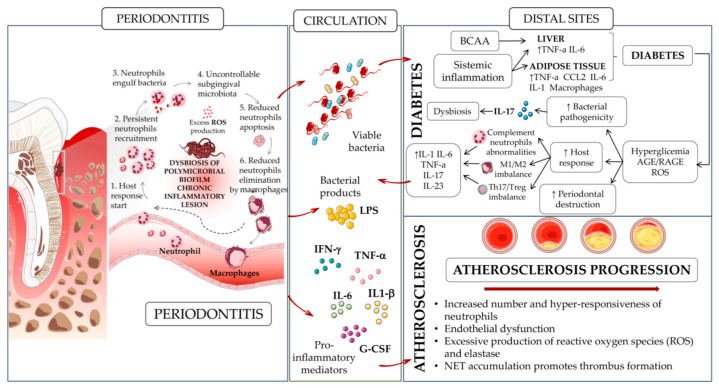
**Neutrophils, periodontitis, and other comorbidities.** This figure illustrates the major reported associations between periodontitis and systemic diseases, with a focus on diabetes and atherosclerosis. Periodontitis is initiated by the ulceration of the gingival epithelium, bacterial invasion, and influx of immune cells, leading to inflammatory damage sustained by ROS generation, the production of different cytokines (IFNγ, IL-6, IL-1β, G-CSF) in periodontal tissues, and the destruction of the supporting alveolar bone. This chronic inflammatory reaction leads to the leakage of bacterial products, host inflammatory factors, and pathogenic oral bacteria into the bloodstream where they are transported to distal tissue sites. Once in systemic circulation, periodontal-derived products have the potential to adversely affect a multitude of systemic diseases, either directly in situ or, indirectly, via the amplification of the systemic inflammatory response. In vivo studies outlined the correlation between classical human periodontal pathogens (*Porphyromonas gingivalis*, *Fusobacterium nucleatum*, and *Prevotella intermedia*), inflammation, and insulin tolerance in periodontitis-related diabetes and diabetes complications, such as inefficient wound-healing and repair. The same studies also showed that, prior to the onset of bone loss and subsequently to HFD, these conditions are associated with the delayed recruitment of neutrophils and monocytes, leading to compromised clearance of bacteria, in association with a peak of different proinflammatory cytokines/chemokines, such as CXCL1, CXCL2, MCP-1, TNFα, IL-1β, and IL-17A. The increased number and hyper-responsiveness of neutrophils in periodontitis may contribute to various stages of atherosclerotic cardiovascular disease (ASCVD) pathology. Elevated neutrophil counts in peripheral blood have been positively correlated with ASCVD risk in periodontitis patients, suggesting a potential role in increasing ASCVD susceptibility. Also, the overactivation of neutrophils through the exacerbated release of proinflammatory cytokines further supports ASCVD increasing endothelial cell damages/dysfunction, excessive ROS production, and NET-mediated generation of thrombi.

Periodontitis and cardiovascular diseases share a biunivocal relationship characterized by complex interactions. The chronic inflammation associated with periodontitis is believed to contribute to systemic inflammation, which can amplify the risk of cardiovascular diseases [94,95]. In this context, inflammatory mediators, including TNF-α, IL-1β, and IL-6 released in response to periodontal infections [68] may foster atherosclerosis and endothelial dysfunction and significantly impact diabetes, thereby exacerbating cardiovascular conditions (Figure 2). Additionally, bacteria originating from periodontal infections can enter the bloodstream [94,95], potentially impacting cardiovascular health through bacterial dissemination and immune responses (Figure 2).

Additionally, the presence of cardiovascular diseases can influence the progression and severity of periodontitis. Cardiovascular conditions such as hypertension and atherosclerosis may compromise blood flow to oral tissues, thereby affecting immune responses and wound healing in the periodontium. Shared risk factors, including smoking, diabetes, and obesity, further accentuate the interconnectedness of these conditions.

Since the link between systemic inflammation and periodontitis/associated cardiovascular diseases has already been addressed in various studies, here we focused on the role of neutrophils in contributing to periodontitis-related comorbidities, namely diabetes and atherosclerosis (Figure 2).

### 4.1. Diabetes

Periodontitis is frequently associated with comorbidities such as diabetes [96,97,98]. Individuals with diabetes have an elevated risk of periodontitis, with a 2–3-fold increase compared to those without diabetes [99]. The degree of glycemic control plays a crucial role in determining this risk [100].

The link between periodontitis and systemic metabolic complications has been largely supported by studies on rodents or lagomorphs recapitulating periodontal disease, such as by ligature-induced periodontitis (LIP) or oral gavage with human periodontal pathogens. These models can recapitulate increased serum levels of acute phase proteins (CRP and serum amyloid A) and inflammatory cytokines (for example, IL-1β and IL-6) occurring during periodontitis [101,102,103] (Figure 2).

Mice receiving a high-fat diet (HFD), following the administration of a combination of three classical human periodontal pathogens (*Porphyromonas gingivalis*, *Fusobacterium nucleatum*, and *Prevotella intermedia*) by oral gavage, show not only localized inflammatory bone loss, but also worsened HFD-induced glucose intolerance and insulin resistance, while increasing the ratio of fat to lean tissue mass [104]. These results were also observed in HFD-fed mice receiving continuous low-rate infusions of LPS from *Porphyromonas gingivalis*, which induced chronic low-grade systemic inflammation [104]. Furthermore, the metabolic activities of periodontal pathogens may potentially exacerbate insulin resistance. In a model of *Porphyromonas gingivalis*-induced periodontitis in HFD-fed mice, infection with the wild-type organism, but not with a mutant deficient in branched-chain amino acid (BCAA) aminotransferase, resulted in elevated serum levels of BCAAs (such as leucine, isoleucine, and valine) and insulin resistance, compared to uninfected HFD-fed mice [105]. This finding aligns with the idea that increased circulating BCAAs are associated with a higher risk of type 2 diabetes (T2DM) [106] (Figure 2).

Periodontitis-associated insulin resistance and subsequent hyperglycemia may trigger the formation of advanced glycation end products (AGEs). This, in turn, can lead to enhanced activation of the proinflammatory receptor for AGE (RAGE), which plays a role in various metabolic disorders, including vascular inflammation [107], atherosclerosis [108], and experimental periodontitis in diabetic mice [108] (Figure 2).

Consistent with other diabetes-related complications, the risk of periodontitis rises with deteriorating glycemic control [109]. A study by Ulvi Kahraman Gursoy et al. analyzed the relationship between neutrophil functions and the severity of periodontitis in obese and/or type 2 diabetic chronic periodontitis patients in a cohort of 39 patients [110]. The authors found no significant differences in terms of age, gingival index, plaque index, percentage of phagocytosis, phagocytic efficiency, and intracellular killing among the patients enrolled [110]. However, the diabetic groups exhibited significantly lower neutrophil chemotaxis compared to the control group, while these results were not significantly affected by obesity for all the parameters evaluated [110].

Periodontitis and impaired wound healing in diabetes are linked to insulin resistance and high blood sugar levels, both of which have been associated with the specific decline in insulin-induced activation of the PI3K/Akt pathway in the gingiva [111]. A very recent study demonstrated that the presence of insulin resistance in the gingiva of mice, either through the selective elimination of insulin receptors in smooth muscle and fibroblasts (SMIRKO mice) or systemic metabolic changes induced in HFD-fed mice, exacerbated alveolar bone loss caused by periodontitis [112]. Prior to the onset of bone loss, these conditions exhibited a delayed recruitment of neutrophils and monocytes, leading to compromised clearance of bacteria compared to their respective control groups [112]. Notably, the expression of CXCL1, CXCL2, MCP-1, TNF-α, IL-1β, and IL-17A displayed delayed peak levels in the gingiva of male SMIRKO and HFD-fed mice when compared to controls (Figure 2). However, targeted overexpression of CXCL1 in the gingiva using an adenovirus normalized the recruitment of neutrophils and monocytes and prevented bone loss in both mouse models of insulin resistance. Mechanistically, insulin augmented the production of CXCL1 in gingival fibroblasts (GFs) from mice and humans in response to bacterial lipopolysaccharide stimulation, acting through the Akt pathway and NF-κB activation. These signaling pathways were found to be diminished in GFs derived from SMIRKO and HFD-fed mice [112]. These findings provide evidence of insulin’s ability to enhance the expression of CXCL1 in response to endotoxins, thus modulating neutrophil recruitment.

As reviewed in this paragraph, most of the studies investigating the bidirectional crosstalk between periodontitis and diabetes (as far as metabolic complications) have largely been conducted using in vitro models, suggesting that a translation into clinical studies is urgent, to identify cellular effectors (such as neutrophils) to be targeted in intervention approaches.

### 4.2. Atherosclerosis

Extensive investigations have examined the impact of periodontitis treatment on cardiovascular outcomes and surrogate markers, as related to atherosclerotic cardiovascular disease (ASCVD). The existing body of evidence consistently demonstrates that patients with periodontitis exhibit a significantly elevated risk of coronary and cerebrovascular events, when compared to healthy control subjects. Cumulative evidence consistently indicates a significant correlation between periodontitis and elevated blood pressure as well as endothelial dysfunction [113,114,115], all relevant risk factors contributing to ASCVD.

Periodontal bacterial and related endotoxins, present during periodontitis, trigger systemic inflammation, as revealed by the increased levels of CRP, TNFα, IL-1β, and IL-6 in the serum of patients [68]. The chronic systemic inflammation state persisting during periodontitis strongly impacts numerous systemic diseases, including ASCVD [116] (Figure 2).

Periodontitis is related to an overactive response by neutrophils [76]. This association could partly be attributed to the idea that oral diseases might affect the activity of hematopoietic tissue, based on the concept of trained immunity [116], understood as a nonspecific memory in innate immune cells that is triggered by previous encounters with infectious or inflammatory stimuli [77,78]. This results in enhanced immune responses to future challenges from similar or different stimuli [77].

The increased number and hyper-responsiveness of neutrophils in periodontitis may contribute to various stages of ASCVD pathology. Elevated neutrophil counts in peripheral blood have been positively correlated with ASCVD risk in periodontitis patients, suggesting a potential role in increasing ASCVD susceptibility [79,80] (Figure 2). Additionally, the consistent presence of endothelial dysfunction in periodontitis patients, a key feature of ASCVD, along with the enhanced production of ROS by neutrophils, induced by periodontitis, can exacerbate the initiation and progression of atherosclerosis (Figure 2). The hyper-responsiveness of neutrophils characterized by an excessive production of neutrophil elastase and ROS in periodontitis patients may contribute to late-stage atherosclerosis by promoting endothelial apoptosis, leading to plaque erosion, fibrous cap thinning, and plaque ruptures. Furthermore, impaired degradation of NETs in the plasma of periodontitis patients could enhance atherosclerosis complications, particularly in postischemic stroke scenarios where NET accumulation promotes thrombus formation and expands stroke volume. Notably, a case–control study revealed that periodontitis was an independent predictor of poor outcomes in postischemic stroke patients [117] (Figure 2).

The existing literature also suggests that hematopoietic stem and progenitor cells (HSPCs) adapted to chronic inflammation (i.e., increased levels of IL-1β, TNF-α, IL-6, type-1 IFN, IFN-γ, G-CSF) contribute to the development of the disease [81,118,119].

In this regard, increased levels of IL-1β have been shown to support myeloid differentiation and self-renewal in HSPCs [9,120,121,122]. TNF-α has been observed to promote the survival of HSPCs and myeloid differentiation, and to induce apoptosis in myeloid progenitor cells [123]. Increased levels of IL-6 enhance myelopoiesis by elevating MPPs and CMPs [124], while an augmented production of Type-1 IFN mediates trained granulopoiesis with the subsequent induction of hyper-responsive neutrophils [45]. Also, increased levels of IFN-γ and G-CSF, both observed in periodontitis-induced chronic inflammation, participate in granulopoiesis [125] and support GMP expansion [126] and granulocyte lineage specification [127], respectively.

Finally, a recent consensus by the European Federation of Periodontology and the World Heart Federation confirmed neutrophil hyper-responsiveness as one of the mechanisms that could explain the epidemiological connection between periodontitis and ASCVD [68,128].

## 5. Conclusions

Neutrophils play a complex role in the pathogenesis of periodontitis, influencing both host defense mechanisms and tissue destruction. Understanding the intricate interplay between neutrophils and their impact on promoting periodontitis and related comorbidities can provide valuable insights for the development of targeted therapeutic strategies for these pathologies, aiming to restore the balance between immune response and tissue homeostasis.

Topics in the literature on therapeutic approaches to periodontitis are strictly limited to the overall inflammatory response, without a specific target on neutrophils. Strategies such as the inhibition of specific neutrophil signaling pathways, modulation of neutrophil recruitment and activation, or promotion of bacterial clearance mechanisms could prevent the persistent release of inflammatory mediators, in turn restoring the balance between the host immune response and tissue homeostasis. In this scenario, developing therapeutic interventions to modulate neutrophil functions in periodontitis holds promise for mitigating tissue destruction and limiting systemic inflammation that significantly contributes to cardiovascular diseases. On the one hand, therapies able to reprogram neutrophils from proinflammatory to reparative neutrophils can be considered: this will help, once the pathogens are eliminated, to restore the vascular and ECM integration of the injured tissue, thus limiting the possible chronic inflammatory/low-grade states that contribute to the development of CVD. Finally, immunotherapy, defined as the ability to both modulate a potential and inhibit an altered immune response, has largely been proposed in the tumor context and is now also being explored for CVD. In line with this, following proper in vitro and in vivo evidence, it would be feasible to propose next-generation immunotherapy able to block the aberrant immune response of neutrophils in periodontitis, with a concomitant redout for periodontitis comorbidities, including CVD.

## Data Availability

Not applicable.

## Abbreviations

AGEs	Advanced Glycation End products
ASCVD	AtheroSclerotic CardioVascular Disease
BCAA	Branched-Chain Amino Acid
BM	Bone Marrow
C/EBPα	CCAAT Enhancer-Binding Protein alpha
CMPs	Common Myeloid Progenitors
CRP	C-Reactive Protein
CXCL-	Chemokine (C-X-C motif) Ligand
CVD	Cardiovascular disease
fMLP	N-Formylmethionyl-leucyl-phenylalanine
G-CSF	Granulocyte Colony-Stimulating Factor
GFs	Gingival Fibroblasts
GMPs	Granulocyte-Monocyte Precursors
HFD	High-Fat Diet
HSC	Hematopoietic Stem Cell
IFN-γ	Interferon-γ
IL-	Interleukin-
LIP	Ligature-Induced Periodontitis
LMPs	Lymphoid and Myeloid Precursors
LPS	LypoPolySaccaride
MCP-1	Monocyte Chemoattractant Protein-1
MMPs	Matrix MetalloProteinases
MPO	MyeloPerOxidase
MPPs	MultiPotent Precursors
NADP-H	Nicotinamide Adenine Dinucleotide Phosphate-H
NE	Neutrophil Elastase
NETs	Neutrophil Extracellular Traps
NF-κB	Nuclear Factor kappa-light-chain-enhancer of activated B cells
PI3K/AKT	Phosphoinositide 3-Kinase Inhibitors
PMA	Phorbol 12-Myristate 13-Acetate
PRRs	Pattern Recognition Receptors
RAGE	Receptor for AGE
ROS	Reactive Oxygen Species
SMIRKO	Smooth Muscle Insulin Receptor Knock Out
TLRs	Toll-like receptors
TNF-α	Tumor Necrosis Factor-α
T2DM	Type 2 Diabetes Morbidity
